# Efficacy of immune checkpoint inhibitors in EGFR-Mutant NSCLC patients with EGFR-TKI resistance: A systematic review and meta-analysis

**DOI:** 10.3389/fphar.2022.926890

**Published:** 2022-08-22

**Authors:** Xiaoyu Qian, Xiaodan Guo, Ting Li, Wei Hu, Lin Zhang, Caisheng Wu, Feng Ye

**Affiliations:** ^1^ Fujian Provincial Key Laboratory of Innovative Drug Target Research, State Key Laboratory of Cellular Stress Biology, School of Pharmaceutical Sciences, Xiamen University, Xiamen, China; ^2^ School of Population Medicine and Public Health, Chinese Academy of Medical Sciences and Peking Union Medical College, Beijing, China; ^3^ Melbourne School of Population and Global Health, The University of Melbourne, Melbourne, VIC, Australia; ^4^ Department of Medical Oncology, Xiamen Key Laboratory of Antitumor Drug Transformation Research, The First Affiliated Hospital of Xiamen University, Xiamen, China; ^5^ Department of Clinical Medicine, Fujian Medical University, Fuzhou, China

**Keywords:** NSCLC, immune checkpoint inhibitors, EGFR mutation, TKIs, meta-analysis

## Abstract

**Background:** Epidermal growth factor receptor (EGFR) mutations are common in patients with non-small-cell lung cancer (NSCLC), particularly in Asian populations. Tyrosine kinase inhibitors (TKIs) are a first-line treatment in patients with mutant EGFR, but their use is often accompanied by drug resistance, which leads to disease progression. Chemotherapy and immunotherapy are the main treatment options after progression. The efficacy of immune checkpoint inhibitors (ICIs) and their combination therapy in patients with EGFR-TKI resistant is not clear. It is thus necessary to evaluate the efficacy of ICIs and ICI-based combination therapies in patients with EGFR-TKI-resistant NSCLC.

**Methods:** We searched for randomized controlled trials (RCTs) comparing ICI therapy alone or in combination versus other therapies using PubMed, the Cochrane Library, Web of Science, EMBASE, MEDLINE, ClinicalTrials.gov, and several international conference databases, from database inception to 10 March 2022. The hazard ratio (HR) and 95% confidence interval (95% CI) for median overall survival (OS) and median progression-free survival (PFS) were evaluated. Odds ratio (OR), risk ratio (RR), and 95% CI were used as effect indicators for objective response rate (ORR) and safety data.

**Results:** Seven eligible RCTs were included in the present meta-analysis. The results showed that neither ICIs nor combination therapy prolonged median OS in EGFR-TKI resistant NSCLC patients (HR = 1.04, 95% CI: 0.84–1.29, *p* = 0.73). However, compared with the control group, the patients treated with ICI-based combination therapy had better PFS (HR = 0.62, 95% CI: 0.45–0.86, *p* = 0.004) and ORR (OR = 1.84, 95% CI: 1.28–2.66, *p* = 0.001).

**Conclusion:** ICI monotherapy did not improve the OS or PFS of NSCLC patients previously treated with EGFR-TKIs, whereas patients treated with ICI-based combination therapy had better PFS compared with those receiving conventional chemotherapy, indicating that this therapy could be offered to patients with EGFR-mutant NSCLC after progression following TKI treatment. There was no significant difference in all-grade treatment-related adverse events (TRAEs) between the combination therapy group and the control group. However, a higher incidence of discontinuation due to TRAEs was observed; this requires attention in future studies. The results of this meta-analysis provide a reference for clinical practice and future trial design.

**PROSPERO registration number:** CRD42021282207

## Introduction

Lung cancer is the main cause of cancer death worldwide. In 2020, the number of deaths due to lung cancer accounted for about 18% of all cancer-related deaths (Cancer Today 2021), and they were mainly concentrated in Europe, America, and Asia. Lung cancer can be divided into small-cell lung cancer and non-small-cell lung cancer (NSCLC). NSCLC is the main type of lung cancer in Western and Asian countries, accounting for about 85% of cases (Thai et al., 2021). Mutations in driving genes are often observed in patients with advanced NSCLC. Mutation of the epidermal growth factor receptor (EGFR) gene is a common mutation type in patients with advanced NSCLC (Sabari et al., 2021), with EGFR mutation rates of 14–20% in European and American populations and about 50% in Asian populations (Dearden et al., 2013; Midha et al., 2015; Zhang et al., 2016).

With the development of targeted therapies, tyrosine kinase inhibitors targeting EGFR (EGFR-TKIs) have become a common treatment for patients with EGFR-mutant advanced NSCLC. TKI treatment has shown significant clinical efficacy compared with traditional chemotherapy. Studies have shown that EGFR-TKIs significantly prolonged the progression-free survival (PFS) and overall survival (OS) of patients (Fukuoka et al., 2011; Zhou et al., 2011; Yang et al., 2015; Greenhalgh et al., 2016; Zhao et al., 2019; Ramalingam et al., 2020), and most of these TKIs have been adopted as standard first-line treatments. However, most patients with EGFR mutation develop drug resistance after receiving TKI treatment for 10–15 months (Wu and Shih, 2018; Wu et al., 2020). The main causes of drug resistance include T790M mutation, c797x mutation, Met amplification, Axl activation, and HER2 amplification (Lim et al., 2018; Westover et al., 2018). For drug-resistant patients with the EGFR T790M mutation, third-generation TKIs are the choice of treatment. However, for resistant patients without EGFR T790M mutation, continuing TKI treatment results in no benefit or even has negative effects (Soria et al., 2015). Traditional pemetrexed-based chemotherapy is still the main treatment for such patients. However, chemotherapy often has serious adverse effects and does not lead to any significant benefit with respect to PFS in patients with EGFR-TKI resistance. Therefore, there is a very limited choice of treatments for advanced NSCLC patients with EGFR mutations and drug resistance.

Immune checkpoint inhibitors (ICIs) are a recently developed type of cancer treatment that has emerged in the past 10 years. Some studies have shown that patients with advanced NSCLC without driver gene mutation can benefit from immunotherapy (Antonia et al., 2019; Mok et al., 2019; Wu et al., 2019; Garassino et al., 2020). Immunotherapy has been used as a first-line treatment for advanced NSCLC. Studies have also shown that activation of the EGFR pathway can induce expression of PD-L1 and related immunosuppressive factors, thereby reshaping the immune microenvironment of tumors (Chen et al., 2015). Mice with EGFR mutation showed a significant response to PD-1 inhibitor treatment (Akbay et al., 2013). EGFR-TKI treatment could also reduce PD-L1 expression and T cell inhibition in patients with NSCLC, resulting in immune enhancement (Hack et al., 2020). This suggests that a combination of a PD-1 inhibitor and an EGFR-TKI may have a better curative effect than TKI alone. However, the combination of PD-1 inhibitors and TKIs as a first-line treatment regimen was shown to have enhanced toxicity with no significant improvement in curative effect (Yang et al., 2019; Oxnard et al., 2020). Therefore, the combination of ICIs and TKIs is not suitable for patients with EGFR-positive NSCLC. Cell experiments have shown that the viability of EGFR-TKI-resistant cells can be decreased by PD-1 inhibitors, which suggests that immunotherapy could be a promising follow-up treatment for patients with EGFR-TKI resistance (Chen et al., 2015).

Whether immunotherapy is suitable for advanced NSCLC patients with EGFR mutation has become a hot-button issue. In studies mentioned above, NSCLC patients with EGFR mutation have generally not benefited from immunotherapy. However, some studies have shown that a small number of patients can benefit from immunotherapy. In addition, there have been few reports on the applications of ICI-related combination therapy in EGFR-TKI-resistant patients. Therefore, the efficacy of ICIs in patients with EGFR-TKI-resistant NSCLC is unclear and needs to be further explored.

The purpose of this meta-analysis was to evaluate the efficacy of ICIs alone and in combination therapies in patients with advanced NSCLC resistant to EGFR-TKI, so as to better address the problems associated with the use of ICIs in such patients.

## Methods

The present systematic review and meta-analysis was conducted following PRISMA guidelines (Page et al., 2021). The protocol has been registered in PROSPERO with the registration number CRD42021282207.

### Data sources and searches

We searched PubMed, the Cochrane Library, Web of Science, EMBASE, and MEDLINE. The retrieval time limit was from the establishment of each database to 10 March 2022. In addition to the above databases, in order to include unpublished studies and obtain more comprehensive relevant research data, we searched the clinical trial registration website (https://clinicaltrials.gov/), conference abstracts of American Society of Clinical Oncology, European Society for Medical Oncology, and World Lung Cancer Conference. The keywords searched included: “non-small cell lung cancer,” “immune checkpoint inhibitors,” “tyrosine kinase inhibitor,” “epithelial growth factor receptor,” and “randomized controlled trial”. The detailed inclusion and exclusion criteria are listed in Table 1.

**TABLE 1:** Inclusion criteria and exclusion criteria.

The eligible studies met the inclusion criteria:
(1) The type of study was RCT.
(2) Phase II and III trials
(3) The intervention arm was patients treated with any PD-1/PD-L1 inhibitor single drug and its combination with immunotherapy, chemotherapy, targeted therapy or antiangiogenic therapy
(4) The control group was patients treated with docetaxel single drug or combined chemotherapy
(5) The subjects were patients with NSCLC diagnosed as EGFR positive by clinicopathological examination, who had previously received EGFR-TKI treatment and experienced disease progression
(6) Studies with available data
Exclusion criteria:
(1) Not randomized clinical trial
(2) Review, meta-analysis, case report and animal experiment
(3) Republished literature
(4) Study without eligible patients
(5) No usable data
(6) Reports from same study sample

### Study selection and data extraction

The main purpose of this meta-analysis was to evaluate the efficacy of ICIs compared with traditional chemotherapy in patients with EGFR-TKI-resistant advanced NSCLC. The subjects were NSCLC patients with EGFR mutation who experienced disease progression after TKI treatment. For each included trial, we extracted the basic characteristics of the study and participants, and retrieved the objective response rate (ORR), hazard ratio (HR), and 95% CI for median OS and median PFS.

Two researchers (Q. X. and G. X.) independently screened the literature, and extracted and cross-checked the data. Discrepancies were resolved by the third researcher. Data types extracted from the trials were: trial phase, authors, year of publication, number of patients, number of patients with EGFR mutation, age of patients, gender of patients, number of smoking patients, treatment comparison, median OS, HR and 95% CI of median OS, median PFS, HR and 95% CI of median PFS, ORR, and safety data. All study periods and follow-up durations were eligible. Titles and abstracts were screened, and the full text of each potentially eligible article was evaluated for final inclusion.

### Data synthesis and analysis

Statistical analysis in this study was performed using the Cochrane Review Manager (RevMan 5.4) software provided by the Cochrane Library Collaboration Network.

The risk of bias of the study was assessed using the Cochrane bias risk assessment tool (Higgins et al., 2011), which includes the following items: random sequence generation, allocation concealment, blinding of participants and personnel, blinding of outcome assessment, incomplete outcome data, selective reporting, and other bias.

For the included studies, HR and 95% CI were used as effect indicators for median OS and median PFS, odds ratio (OR) and 95% CI were used as effect indicators for ORR, and risk ratio (RR) and 95% CI were used for safety data. I^2^ index and q-statistics were used to evaluate the heterogeneity among studies. If I^2^<50%, *p* > 0.05, indicating that the research results may be homogeneous, the fixed effect model is used for analysis. In contrast, when I^2^>50%, *p* < 0.05, indicating substantial heterogeneity, the source of heterogeneity should be analyzed. If the heterogeneity still exists, the random effect model should be used.

## Results

### Search results

**FIGURE 1:**
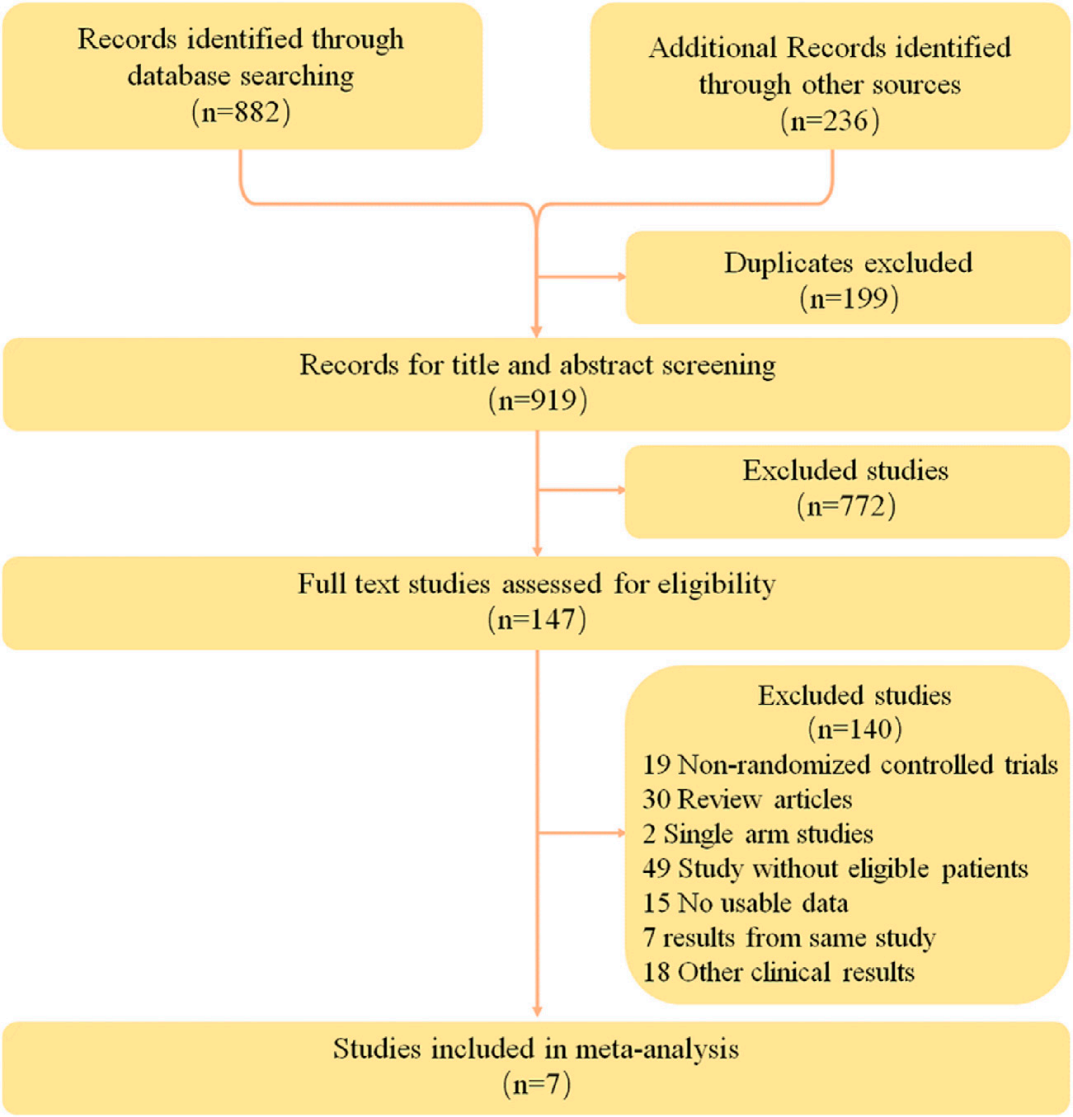
PRISMA Flow Diagram of Study Selection for this Meta-Analysis.

A total of 1118 relevant studies were retrieved according to the retrieval strategy. The literature was screened according to the exclusion criteria, and finally seven studies (Borghaei et al., 2015; Herbst et al., 2016; Rittmeyer et al., 2017; Arrieta et al., 2020; Hayashi et al., 2022; Lu et al., 2022; Nogami et al., 2022) were included. These studies involved a total of 4,292 patients, including 2,453 in the intervention groups and 1839 in the control groups. Data from one study (Lu et al., 2022) was obtained from relevant conference abstracts. The literature retrieval results and flow chart are shown in Figure 1, and the basic information of the included studies is shown in Table 2.

**TABLE 2:** Characteristics of the studies included in the meta-analysis.

Study (year)	Phase	Author	Sample size	Female	Non-smokers (%)	EGFR mutation	Intervention arm (n)	Control arm (n)	Median OS (mo)	Median PFS (mo)	ORR (%)	References
Checkmate057 (2015)	III	Borghaei, H. et al	582	263	20	82	Nivolumab (44)	Docetaxel (38)	12.2 vs. 9.4	2.3 vs. 4.2	NG	Borghaei et al. (2015)
KEYNOTE-010 (2016)	II/III	Herbst, R. S. et al	1034	400	18	86	Pembrolizumab (60)	Docetaxel (26)	10.4^a^ vs. 12.7^b^ vs. 8.5	5^a^ vs. 5.2^b^ vs. 4.1	NG	Herbst et al. (2016)
OAK (2017)	III	Rittmeyer, A. et al	850	330	18	85	Atezolizumab (42)	Docetaxel (43)	13.8 vs. 9.6	NG	NG	Rittmeyer et al. (2017)
PROLUNG (2020)	II	Arrieta O et al	78	50	43	25	pembrolizumab + docetaxel (12)	Docetaxel (13)	8.3 vs. 13.1	6.8 vs. 3.5	58.3 vs.23.1	Arrieta et al. (2020)
IMpower150 (2022)	III	Nogami, N et al	1202	482	20	123	A:Atezolizumab + Bevacizumab + Carboplatin + Paclitaxel (34)	Bevacizumab + Carboplatin + Paclitaxel (44)	26.1 vs. 21.4 vs. 20.3	10.2 vs. 6.9 vs. 7.1	70.6 vs. 35.6 vs. 41.9	Nogami et al. (2022)
							B:Atezolizumab + Carboplatin + Paclitaxel (45)					
ORIENT-31 (2022)	III	S.Lu et al	444	261	70	444	A:Sintilimab + IBI305 + Pemetrexed + Cisplatin (148)	Placebo1+Placebo2+Pemetrexed + Cisplatin (151)	NG	6.9 vs. 5.6 vs. 4.3	43.9 vs. 33.1 vs. 25.2	Lu et al. (2022)
							B:Sintilimab + Placebo2+Pemetrexed + Cisplatin (145)					
WJOG8515L (2022)	II	Hidetoshi Hayashi et al	102	59	56	102	Nivolumab (52)	Carboplatin + Pemetrexed (50)	20.7 vs. 19.9	1.7 vs. 5.6	9.6 vs. 36.0	Hayashi et al. (2022)

a Pembrolizumab, 2 mg/kg arm.

b Pembrolizumab, 10 mg/kg arm.

NG, not given; IBI305, Biosimilar of Bevacizumab.

Of the included patients, a total of 902 (21.01%) had known EGFR mutations and had received previous TKI treatment, with disease progression during or after treatment. Of the seven therapeutic regimens, three (Arrieta et al., 2020; Lu et al., 2022; Nogami et al., 2022) were combination therapy, and four (Borghaei et al., 2015; Herbst et al., 2016; Rittmeyer et al., 2017; Hayashi et al., 2022) were ICI monotherapy. In the control group, three studies (Borghaei et al., 2015; Herbst et al., 2016; Rittmeyer et al., 2017) used docetaxel monotherapy, one study (Hayashi et al., 2022) used carboplatin plus pemetrexed, one study (Lu et al., 2022) used placebo plus pemetrexed and cisplatin, and one study (Nogami et al., 2022) used bevacizumab plus carboplatin and paclitaxel.

### Analysis of median OS

**FIGURE 2:**
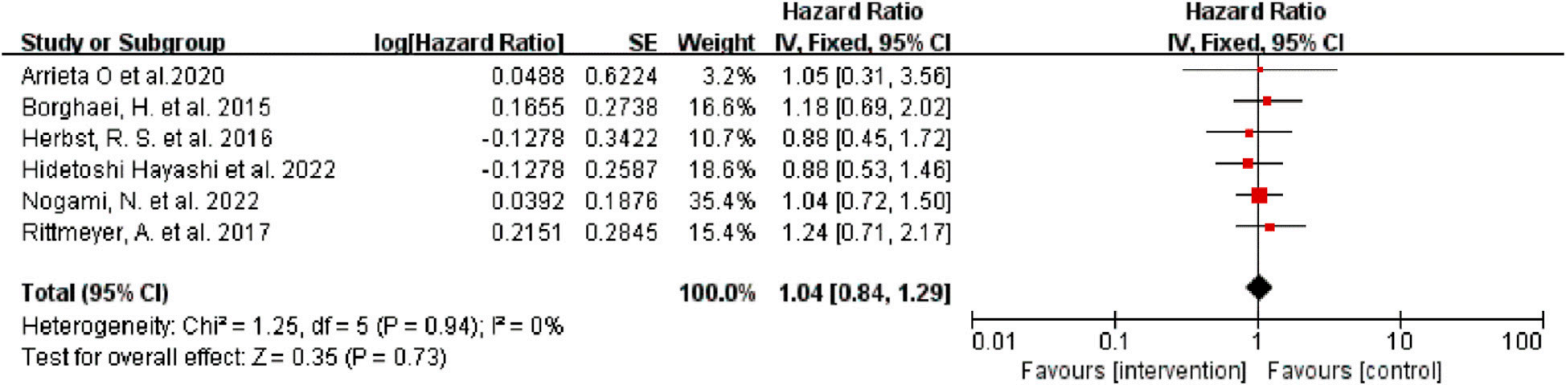
Forest Plot of Hazard Ratios Comparing OS in Patients Who Received Immune Checkpoint Inhibitors vs. Other Therapeutics.

Six of the seven studies reported the median OS of patients, and one did not. According to the results of the heterogeneity test (I^2^ = 0, *p* = 0.95) the heterogeneity among the studies was low. The results were pooled and analyzed by a fixed effect model. The results showed that there was no significant difference in median OS between the intervention group and the control group (HR = 1.04, 95% CI: 0.84–1.29, *p* = 0.73) (Figure 2), regardless of whether the intervention was monotherapy or combination therapy.

### Analysis of median PFS

**FIGURE 3:**
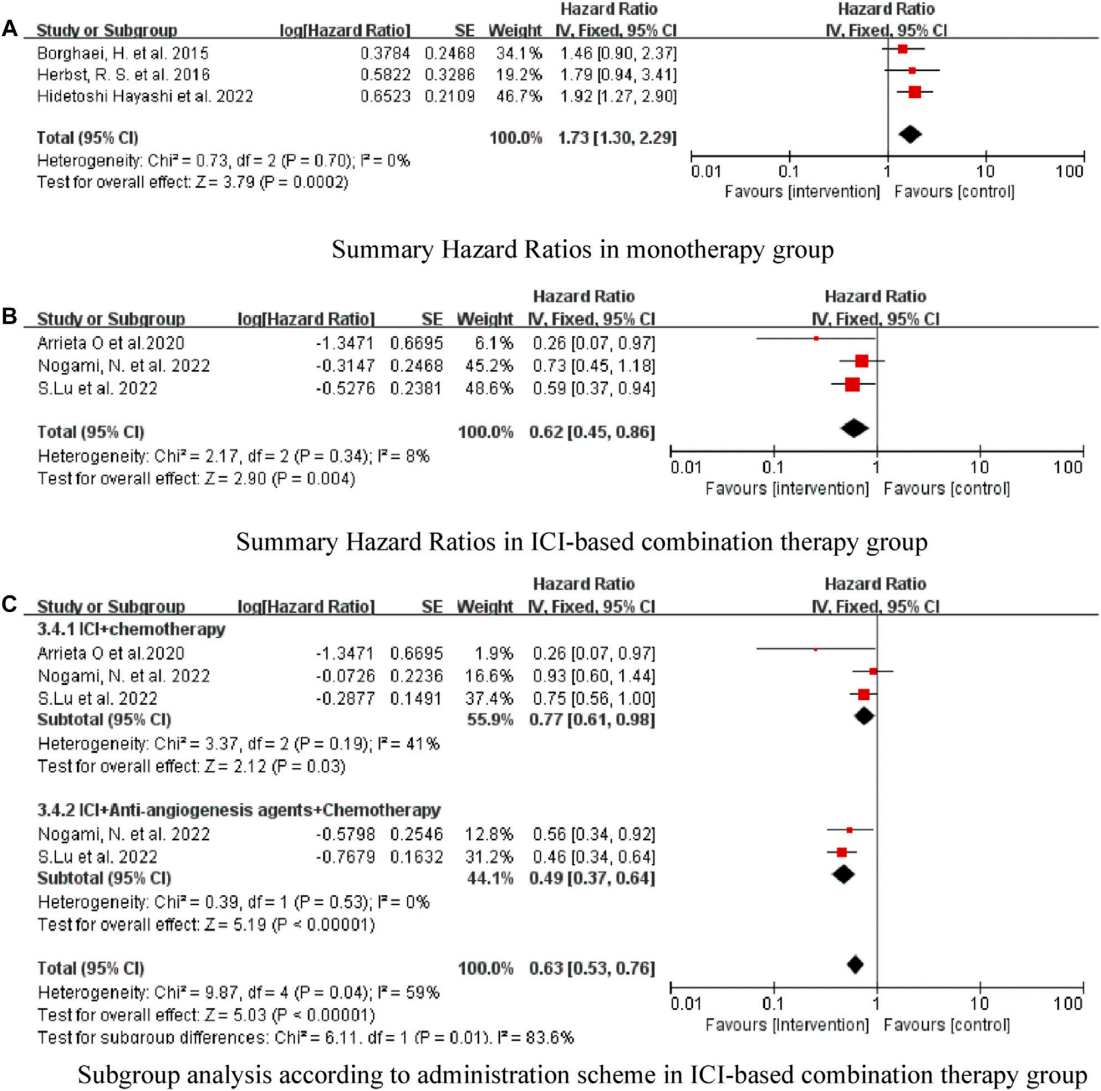
Forest Plot of HR Comparing PFS in Patients Who Received ICIs vs. Other Therapeutics. **(A)** Summary Hazard Ratios in monotherapy group. **(B)** Summary Hazard Ratios in ICI-based combination therapy group. **(C)** Subgroup analysis according to administration scheme in ICI-based combination therapy group.

Six of the seven studies reported the median PFS of patients, and one did not. In the monotherapy group, there was no improvement in PFS, and traditional chemotherapy showed better efficacy (HR = 1.73, 95% CI:1.30–2.29, *p* = 0.0002) (Figure 3A). The pooled results showed that ICI-based combination therapy could prolong PFS, with a pooled HR of 0.62 (95% CI: 0.45–0.86, *p* = 0.004) (Figure 3B). Subgroup analysis according to the specific administration scheme showed that ICI plus chemotherapy was beneficial with respect to PFS. Notably, the effect of ICI plus anti-angiogenesis agents plus chemotherapy was better than ICI plus chemotherapy (HR = 0.49, 95% CI: 0.37–0.64, *p* < 0.00001) (Figure 3C).

### Analysis of ORR

**FIGURE 4:**
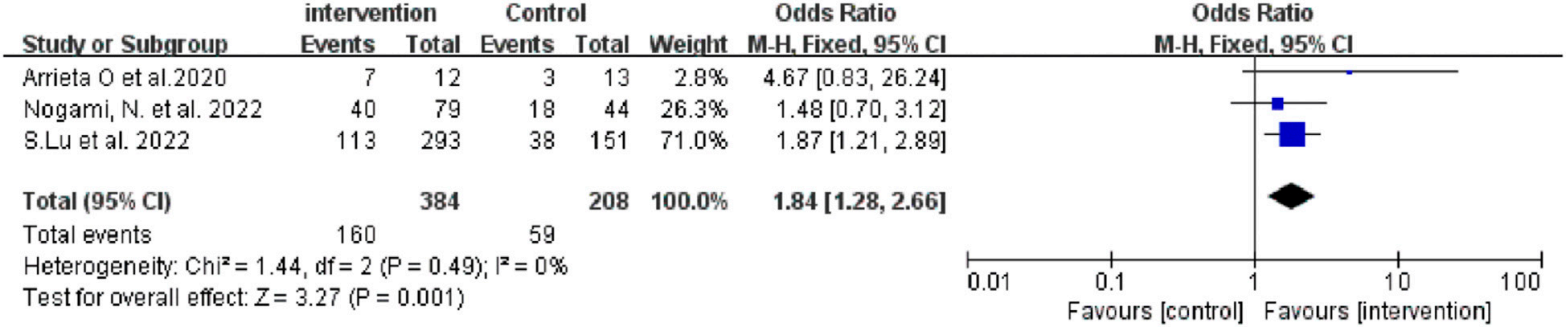
Forest Plot of OR Comparing ORR in Patients Who Received ICI-based combination therapy vs. Other Therapeutics.

Four of the seven studies reported the median PFS of patients, and three did not. Only one article reported ORR in the monotherapy group, so it was impossible to calculate the ORR of monotherapy. We only conducted a meta-analysis on the ORR of the combination group (Figure 4).

The results showed that ICI-based combination therapy was associated with better ORR than the control treatment (OR = 1.84, 95% CI: 1.28–2.66, *p* = 0.001). Subgroup analysis showed that ICI plus chemistry was beneficial with respect to ORR, but this benefit was not statistically significant. ICI plus anti-angiogenesis agents plus chemotherapy had better efficacy than ICI plus chemotherapy (Table 3).

**TABLE 3:** Subgroup analysis.

Outcome indicator	Treatment	Subgroup	No. of studies	Test for overall effect			Heterogeneity	
				HR/OR	95% CI	*p* value	I^2^	*p* value
PFS	Combination therapy	Total	3	0.62	0.45–0.86	0.004	8%	0.34
		ICI + Chemotherapy	3	0.77	0.61–0.98	0.03	41%	0.19
		ICI + Anti-angiogenesis agents + Chemotherapy	2	0.49	0.37–0.64	<0.00001	0%	0.53
OS	Monotherapy	Total	4	1.04	0.79–1.37	0.79	0%	0.74
	Combination therapy	Total	2	1.04	0.73–1.48	0.82	0%	0.99
		ICI + Chemotherapy	2	1.14	0.73–1.80	0.56	0%	0.88
		ICI + Anti-angiogenesis agents + Chemotherapy	1	0.91	0.53–1.59	0.73	-	-
ORR	Combination therapy	Total	3	1.84	1.28–2.66	0.001	0%	0.49
		ICI + Chemotherapy	3	1.36	0.90–2.07	0.15	44%	0.17
		ICI + Anti-angiogenesis agents + Chemotherapy	2	2.53	1.64–3.91	<0.00001	0%	0.47
PFS	Monotherapy	PD-L1 tumor proportion≥1%	2	1.84	1.11–3.04	0.02	0%	0.89
		PD-L1 tumor proportion<1%	1	1.67	0.90–3.10	0.10	-	-
	Combination therapy	PD-L1 tumor proportion (-)	1	0.91	0.63–1.32	0.62	-	-
		PD-L1 tumor proportion≥1%	1	0.66	0.46–0.94	0.02	-	-
		PD-L1 tumor proportion≥50%	1	0.46	0.34–0.84	0.02	-	-
OS	Monotherapy	PD-L1 tumor proportion≥1%	1	0.88	0.45–1.70	0.71	-	-
	Combination therapy	PD-L1 tumor proportion (-)	1	0.90	0.75–1.08	0.27	-	-
		PD-L1 tumor proportion≥1%	1	0.75	0.59–0.95	0.02	-	-
		PD-L1 tumor proportion≥50%	1	0.71	0.51–0.99	0.04	-	-

### Analysis of safety

**FIGURE 5:**
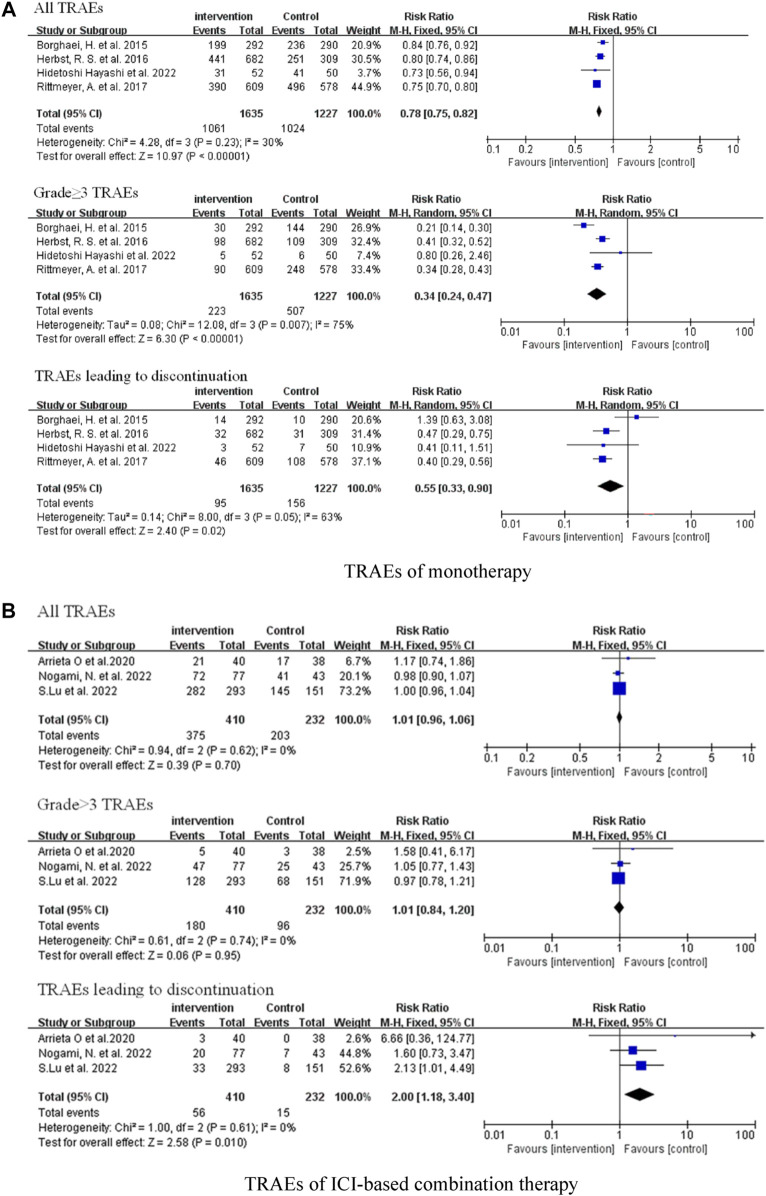
Forest Plot of Overall Comparison of TRAEs. **(A)** TRAEs of monotherapy. **(B)** TRAEs of ICI-based combination therapy.

We investigated all-grade treatment-related adverse events (TRAEs), grade ≥3 TRAEs, and TRAEs leading to discontinuation of included studies. Compared with traditional chemotherapy, ICI monotherapy did not cause more serious adverse reactions, whether all-grade TRAEs (RR = 0.78, 95% CI: 0.75–0.82, *p* < 0.00001), grade ≥3 TRAEs (RR = 0.34, 95% CI: 0.24–0.47, *p* < 0.00001), or TRAEs leading to discontinuation (RR = 0.55, 95% CI: 0.33–0.90, *p* = 0.02) (Figure 5A). There was no significant difference in the frequency of all-grade TRAEs (RR = 1.01, 95% CI: 0.96–1.06, *p* = 0.70) and grade ≥3 TRAEs (RR = 1.01, 95% CI: 0.84–1.20, *p* = 0.95) between the combination therapy and chemotherapy groups; however, combination therapy was associated with more TRAEs leading to discontinuation (RR = 2.00, 95% CI: 1.18–3.40, *p* = 0.01) (Figure 5B).

### Heterogeneity and bias of included studies

**FIGURE 6:**
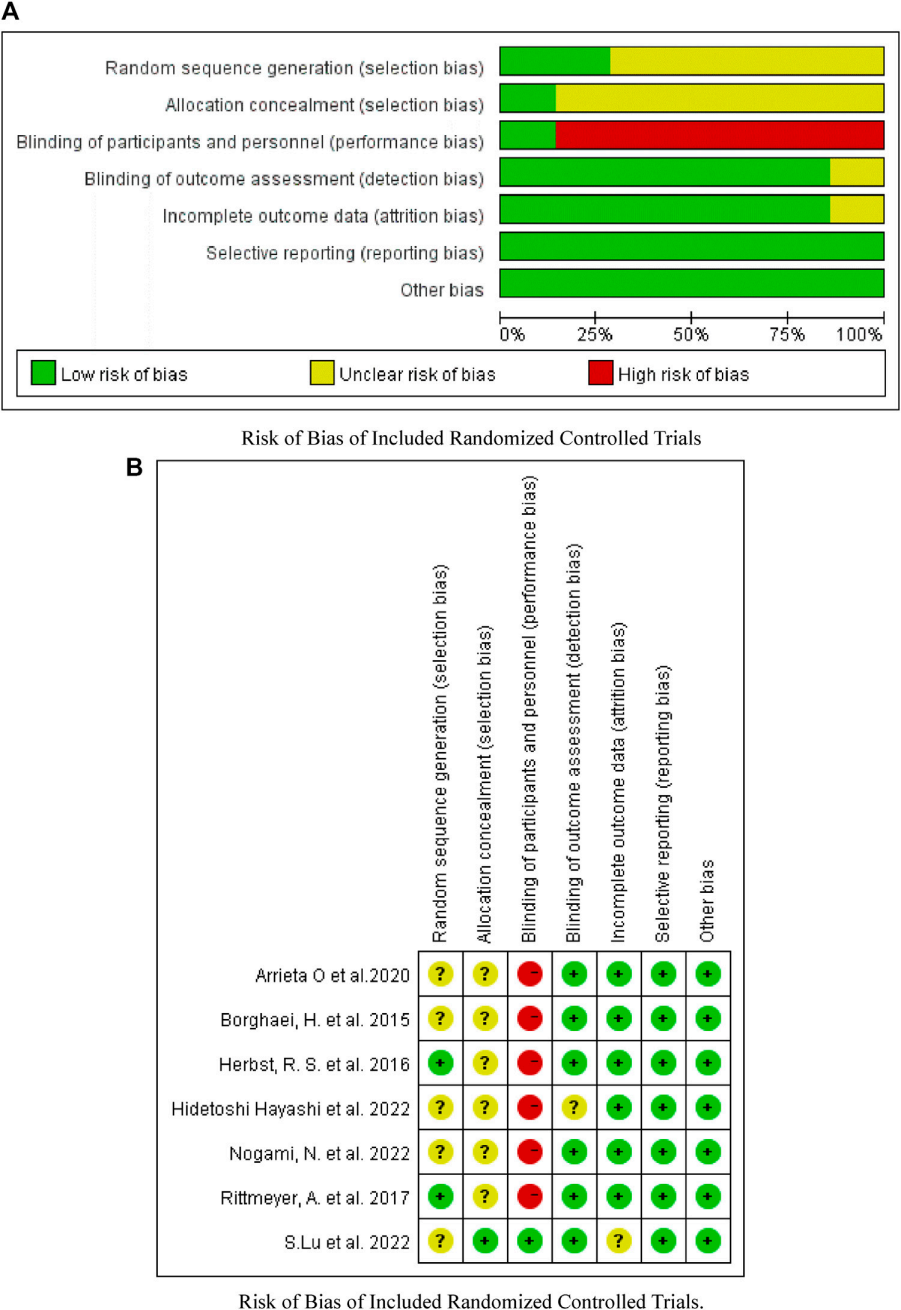
Assessment of bias risk. **(A)** Risk of Bias of Included Randomized Controlled Trials. **(B)** Risk of Bias of Included Randomized Controlled Trials.

As shown in Figures 2–4, there was low heterogeneity with respect to median OS (I^2^ = 0%, *p* = 0.94), median PFS (I^2^ = 0%, *p* = 0.70 for monotherapy; I^2^ = 8%, *p* = 0.34 for combination therapy) and ORR (I^2^ = 0%, *p* = 0.49) among the included studies. The results obtained with the Cochrane risk-of-bias assessment tool for the seven enrolled RCTs are shown in Figure 6; Table 4.

**TABLE 4:** Risk of bias of included randomized controlled trials.

Author	Year	Random sequence generation	Allocation concealment	Blinding of participants and personnel	Blinding of outcome assessment	Incomplete outcome data	Selective reporting	Other bias
Borghaei, H. et al	2015	Unclear	Unclear	High	Low	Low	Low	Low
Herbst, R. S. et al	2016	Low	Unclear	High	Low	Low	Low	Low
Rittmeyer, A. et al	2017	Low	Unclear	High	Low	Low	Low	Low
Arrieta O et al	2020	Unclear	Unclear	High	Low	Low	Low	Low
Nogami, N et al	2022	Unclear	Unclear	High	Low	Low	Low	Low
S.Lu et al	2022	Unclear	Low	Low	Low	Unclear	Low	Low
Hidetoshi Hayashi et al	2022	Unclear	Unclear	High	Unclear	Low	Low	Low

## Discussion

This meta-analysis showed that in NSCLC patients with disease progression or recurrence after TKIs treatment, immunotherapy as second-line or third-line treatment confers no benefit with respect to OS and PFS compared with chemotherapy. However, chemotherapy resulted in better PFS in the patients with TKI treatment progress, consistent with previous research results (Lee et al., 2017; Cavanna et al., 2019). ICI-based combination therapy showed better results as a second-line or third-line treatment with respect to PFS. A combination of a PD-1 inhibitor, antiangiogenic agents, and chemotherapeutic agents significantly improved the PFS and ORR of patients with EGFR mutation, suggesting that combination therapy may be an option for EGFR-TKI-resistant patients. Although combined immunotherapy and chemotherapy performed well in terms of PFS, there was no significant advantage in the ORR analysis. Although the analysis results tended to be combined therapy, it was not statistically significant. Owing to the limited number of studies included, further examination is needed in the future to determine the efficiency of the combination of immunotherapy and chemotherapy. Recent research has also investigated the efficiency of combinations of immunotherapy and chemotherapy. The ORR of toripalimab combined with carboplatin/pemetrexed in an intention-to-treat population was 54.8% (Zhang et al., 2019), and that of tislelizumab combined with albumin paclitaxel/carboplatin was 59.4% (Han et al., 2021). Immunotherapy combined with chemotherapy showed an advantage in terms of ORR in EGFR-mutant patients, but the final data remains to be investigated.

In order to further explore the efficacy of combined therapy, we systematically searched observational studies and found only three relevant studies. One of these studies compared the efficacy of chemotherapy plus ICIs with and without anti-angiogenic drugs and chemotherapy alone (Deng et al., 2021). However, the other two studies were comparisons between combination therapy and ICI monotherapy (Tian et al., 2021; Bai et al., 2022); these studies were inconsistent with the requirements of our control group and thus no further data analysis could be carried out. The results of Deng et al. showed that the combined treatment could prolong median PFS by 2.9 months (HR = 0.22; 95% CI: 0.05–0.93; *p* = 0.039), and the ORR was better (50% in combination group vs. 22% in chemotherapy group). Tian et al. found that the ICI-based combination therapy was better than ICI monotherapy, with median PFS values of five and 2.2 months and median OS values of 14.4 and 7 months, respectively. Bai et al. found that the ICI-based combination therapy prolonged the survival of patients, especially the regimen of immunotherapy plus chemotherapy plus antiangiogenetic agents. All of these studies showed that combination therapy was effective in patients with previous TKI progression, and the combination of immunotherapy plus chemotherapy plus antiangiogenetic agents was the most effective treatment.

In terms of safety, ICI monotherapy did not show more serious toxicity overall compared with chemotherapy. There was no significant difference in frequency of all-grade TRAEs and grade ≥3 TRAEs between the combination treatment group and the control group. However, the incidence of adverse events leading to discontinuation in the combined treatment group was higher than that in the control group. The health status of patients should thus be closely monitored. The TRAEs of grade 3 or higher in the combination therapy group were mainly neutropenia, anemia, hypertension, and leucopenia, and no additional safety signals were found.

Although studies have shown that PD-1/L1 inhibitors represent a promising treatment for patients with EGFR-TKI-resistant NSCLC, according to our meta-analysis, ICI monotherapy did not have a significant effect. The formation of blood vessels around the tumor is a possible reason for the lack of effectiveness of immunotherapy. Such neovascularization around the tumor forms a physical barrier that prevents the infiltration of immune cells into the tumor tissue. Tumor vascular endothelial growth factor (VEGF) also induces FasL expression and thus triggers T cell apoptosis; therefore, even if the ICI can activate more immune cells, it is difficult for these to have a considerable effect. Antiangiogenic agents can induce tumor vascular normalization by inhibiting VEGF, weakening the physical barrier of tumor angiogenesis, increasing the infiltration of immune cells, and further activating immune effector cells under the action of ICI, thereby improving the efficacy of immunotherapy. The infiltration of more immune effector cells promotes the release of interferon γ and vascular remodeling, which in turn improves the efficacy of angiogenic agents and forms a virtuous circle (Motz et al., 2014; Tian et al., 2017). Further, the addition of chemotherapy can accelerate the apoptosis of tumor cells and promote the release of related antigens, as well as reducing the number of immune suppressor cells, thereby regulating the tumor immune microenvironment together with antiangiogenic agents (Chen and Mellman, 2013); this is a possible mechanism underlying the effects of the combination of ICIs with antiangiogenic agents and chemotherapy.

A major advantage of this study is that it included data from all the latest relevant trials, including three outcome indicators from seven studies; it also included data on immune-related combination therapy for the first time. The study population was only composed of EGFR-mutant NSCLC patients who had received previous TKI treatment, which ensured the homogeneity of the study population to a certain extent. The results of this meta-analysis suggest that combination therapy may have a better therapeutic effect than immune monotherapy; the combination of ICIs and antiangiogenic agents in particular deserves further attention.

There were many limitations of this meta-analysis. Only a limited number of trials were included in this study. As the subject matter has only become a research hotspot in recent years, many trials are still in progress. Several intention-to-treat studies have shown that ICI monotherapy is not effective, decreasing the research focus on ICIs monotherapy. Most current research focuses on ICI combination therapy ICIs in EGFR-TKI-resistant patients; in particular, ICIs combined with chemotherapy and ICIs combined with antiangiogenic agents have become a research hotspot. We have summarized the relevant RCTs in progress (Table 5) to provide guidance for follow-up research. Only multi-arm RCTs were included in this study, and many single-arm trials were excluded, which led to a small sample size and limited data. In addition, it is undeniable that the therapeutic effect of ICIs is affected by PD-L1 tumor proportion. In the included studies, only one study reported OS in people with PD-L1 tumor proportion ≥1% in the monotherapy group. The results showed that the effect of ICI was still not as good as that of docetaxel. In the combination therapy group, one study reported that there was no significant difference between combined therapy and chemotherapy in a population without PD-L1 expression. When the PD-L1 tumor proportion score was ≥1%, the results were biased towards combination therapy, regardless of whether PD-L1 expression was high or low. Two studies reported PFS in people with PD-L1 expression ≥1% in the monotherapy group; chemotherapy still worked better than ICI monotherapy. In the combination group, one study reported data related to PD-L1 expression; the results were the same as those for OS (Table 3). In the phase II single-arm ATLANTIC study, NSCLC patients were divided according to their PD-L1 expression status (Garassino et al., 2018). In the EGFR mutation subgroup, the median OS of the population with PD-L1 expression <25% treated with durvalumab was 9.9 months (HR 5.9–10.8), whereas that of the population with PD-L1 expression ≥25% was 16.1 months (HR 6.2–33.2). Although the ORR of the EGFR mutation subgroup was lower than that of the total population, patients with higher PD-L1 expression had better median OS and higher ORR. In this meta-analysis, the EGFR mutation type, smoking status, and number of TKI treatment lines received in the past were unknown; investigation of PD-L1 expression was also limited. Although these factors may affect the treatment effect, it was impossible to investigate their impact owing to the limited reports in the literature. Owing to these limitations, more clinical trials are needed in the future to study relevant problems and obtain more detailed data for further summary, so as to elucidate the therapeutic effect of ICIs in NSCLC patients with EGFR mutation.

**TABLE 5:** Ongoing randomized controlled trials of immune checkpoint inhibitors in EGFR-TKI-resistant NSCLC.

Study	Phase	Intervention arm	Control arm	Actual study start date	Outcome measures	Estimated study completion date
KEYNOTE-789	III	Pembrolizumab + Pemetrexed + Carboplatin + Cisplatin	Placebo + Pemetrexed + Carboplatin + Cisplatin	29 June 2018	PFS, OS	15 June 2023
CheckMate722	III	Nivolumab + Pemetrexed + Cisplatin + Carboplatin	Pemetrexed + Cisplatin + Carboplatin	17 March 2017	PFS	15 July 2022
		Nivolumab + Ipilimumab				
ATTLAS	III	Atezolizumab + Bevacizumab + Carboplatin + Paclitaxel	Pemetrexed + Carboplatin or Cisplatin	27 August 2019	PFS	31 December 2022
ETOP 15-19 ABC-lung	II	Atezolizumab + Bevacizumab + Carboplatin + Paclitaxel	Atezolizumab + Bevacizumab + Pemetrexed	1 July 2020	PFS	31 December 2022
TH-138	II	Carboplatin + Pemetrexed + Bevacizumab + Atezolizumab	Carboplatin + Pemetrexed + Bevacizumab	4 September 2019	PFS	January 2024
NCT03091491	II	Nivolumab and Ipilimumab	Nivolumab	7 April 2017	ORR	December 2021

In conclusion, compared with chemotherapy, ICI monotherapy as a second-line or third-line treatment did not significantly improve the survival of patients with EGFR-TKI resistant NSCLC according to the data analyzed. For patients with EGFR mutation, based on the reports included in this study, the expression state of PD-L1 does not have a significant impact on the effect of monotherapy. In PD-L1+ patients, combination therapy has shown good efficacy. Further investigation of the mechanisms of PD-L1 expression and drug resistance will help to guide subsequent immunotherapy. Combination therapy seems to play a better role than monotherapy; however, according to the results of our analysis, the efficacy of ICI combined with chemotherapy needs further study. The combination of ICIs with chemotherapy or antiangiogenic agents showed considerable efficacy. Owing to the limited sample size, the therapeutic value of this combination treatment scheme needs to be verified using more clinical trial data.

ICI-based combination therapy could be used as a follow-up treatment option for patients with EGFR-TKI-resistant NSCLC, especially the combination of ICIs with chemotherapy or antiangiogenic agents. There was no significant difference in all-grade TRAEs between the combination group and the control group, but it should be emphasized that combination therapy was related to a higher incidence of TRAEs leading to discontinuation, which requires close attention. Furthermore, the treatment cost of the combination of three agents is relatively high, which will lead to fewer patients taking it as a follow-up treatment option. How to optimize the treatment strategy of ICI combination therapy to reduce toxicity and economic burden is a difficult problem to be overcome; more and larger-scale research is needed. Finally, it is worth mentioning that the reliability of meta-analysis results and their applicability in clinical practice depend on critical thinking and objective judgment.

## Data Availability

The original contributions presented in the study are included in the article/supplementary material, further inquiries can be directed to the corresponding authors.
